# Near Work Related Parameters and Myopia in Chinese Children: the Anyang Childhood Eye Study

**DOI:** 10.1371/journal.pone.0134514

**Published:** 2015-08-05

**Authors:** Shi-Ming Li, Si-Yuan Li, Meng-Tian Kang, Yuehua Zhou, Luo-Ru Liu, He Li, Yi-Peng Wang, Si-Yan Zhan, Bamini Gopinath, Paul Mitchell, Ningli Wang, Anyang Childhood Eye Study Group

**Affiliations:** 1 Beijing Tongren Eye Center, Beijing Tongren Hospital, Beijing Ophthalmology Visual Science Key Lab, Beijing Institute of Ophthalmology, Capital Medical University, Beijing, China; 2 Anyang Eye Hospital, Anyang, Henan Province, China; 3 Department of Epidemiology and Health Statistics, Peking University School of Public Health, Beijing, China; 4 Centre for Vision Research, Department of Ophthalmology and Westmead Millennium Institute, University of Sydney, Sydney, Australia; Medical College of Soochow University, CHINA

## Abstract

**Purpose:**

To examine the associations of near work related parameters with spherical equivalent refraction and axial length in Chinese children.

**Methods:**

A total of 1770 grade 7 students with mean age of 12.7 years were examined with cycloplegic autorefraction and axial length. Questions were asked regarding time spent in near work and outdoors per day, and near work related parameters.

**Results:**

Multivariate models revealed the following associations with greater odds of myopia: continuous reading (> 45min), odds ratio [OR], 1.4; 95% confidence interval [CI], 1.1-1.8; close television viewing distance (≤ 3m), OR, 1.7; 95% CI, 1.2-2.3; head tilt when writing, OR, 1.3; 95% CI, 1.1-1.7, and desk lighting using fluorescent vs. incandescent lamp, OR, 1.5; 95% CI, 1.2-2.0. These factors, together with close reading distance and close nib-to-fingertip distance were significantly associated with greater myopia (*P*<0.01). Among near work activities, only reading more books for pleasure was significantly associated with greater myopia (*P*=0.03). Television viewing distance (≤ 3 m), fluorescent desk light, close reading distance (≤20 cm) and close nib-to-fingertip distance (≤ 2 cm) were significantly associated with longer axial length (*P*<0.01). Reading distance, desk light, and reading books for pleasure had significant interaction effects with parental myopia.

**Conclusions:**

Continuous reading, close distances of reading, television viewing and nib-to-fingertip, head tilt when writing, reading more books for pleasure and use of fluorescent desk light were significantly associated with myopia in 12-year-old Chinese children, which indicates that visual behaviors and environments may be important factors mediating the effects of near work on myopia.

## Introduction

Associations of myopia with education have been consistently reported in Eskimo populations [1], Chinese fisherman in Hong Kong [2], and Singapore military conscripts [3]. It is therefore a commonly-held view that the remarkable rise in myopia prevalence in urban East Asia might be associated with increasing intensity of education. Near work has long been considered as a possible mediator for this association through increased accommodative demand [4]. However, the evidence for near work as a risk factor for myopia remains controversial [5]. Some studies have reported that greater near work exposure was associated with both higher prevalence [6] and incidence [7] of myopia in children. On the contrary, some studies found non-significant effects of near work on myopia status [8], myopia incidence [9], and myopia progression [10].

During near work activities such as reading and writing, accommodation increases and was assumed to mediate the association of myopia development with near work [11]. However, the evidence of this mediation from both human[6,8] and animal[12] studies was not strong. Animal studies showed that accommodation might not play an important role in myopia development [12]. Accommodative lag, which was found to be higher in myopic children than in emmetropic children [13], causes hyperopic defocus on both central and peripheral retina that might stimulate eye growth. Evidence of the association between accommodative lag and myopia, however, is also controversial [14,15]. Therefore, the intermediate link between myopia and near work remains unclear.

Recently, it was found that myopia was significantly associated with continuous reading and close reading distance in Australian Caucasian children [16]. These indicate that the intensity rather than the total duration of near work may play a role in myopia development. However, these associations were not found in their 238 East Asian children and have not been demonstrated in other populations such as children in mainland China [16]. It is therefore necessary to explore whether these near work related parameters are associated with myopia in a larger number of Chinese children. Moreover, Wang et al. found that Chinese children had closer reading distances of 16~28 cm and greater angle of head tilt when writing than in other samples [17]. Hartwig et al. reported that head posture and movement during reading may differ in myopic progression [18]. These parameters have not been evaluated in previous study in Australia either.

In the present study, we sought to evaluate the cross-sectional associations of myopia with near work related parameters including continuous reading, reading distance, television viewing distance, nib-to fingertip distance and head tilt when writing, desk lighting, and use of night-lights in a large sample of grade 7 Chinese children.

## Materials and Methods

### Study Population

This study was approved by the Ethics Committee of Beijing Tongue Hospital, Capital Medical University, and adhered to the tenets of the Declaration of Helsinki. Informed written consent was obtained from at least one parent of each child. Verbal assent was also obtained from all children. The methodology and baseline data from the Anyang Childhood Eye Study (ACES) have been reported elsewhere [19]. During the period from October 2011 to December 2011, 2267 grade 7 students aged 12.7 (range, 10~15) years were examined by ophthalmologists and trained medical professionals with a response rate of 95.9% [19].

### Examinations

Cycloplegic autorefraction was performed 30 minutes after one drop of topical anesthetic agent (Alcaine, Alcon), 2 drops of 1% cyclopentolate (Alcon) and 1 drop of 0.5% tropicamide (Mydrin P, Santen, Japan) at 5-minute intervals [19]. Three measurements were automatically performed and averaged (HRK-7000A, Huvitz, Korea). The IOL Master (Carl Zeiss Meditec AG, Jena, Germany) was used to measure axial length.

### Questionnaire Data

An interviewer-administered questionnaire was administrated to parents to identify the proportion with myopia[8] and near work related parameters (S1 file). These parameters were mainly focused on the longest duration, distance, angle of viewing and lighting for all kinds of near work activity, which included children’s duration of continuous reading, reading distances, an observed head tilt and nib-to-fingertip distance when writing, type of desk light used for reading, use of night-lights and television viewing distance. There was an option of ‘not sure’ for these parameters. Time (hours/day) engaged in near work and outdoor activities were estimated using an interviewer-administrated questionnaire to the children. Near work includes school homework, reading books for pleasure, using a computer, playing console games, drawing, painting, writing, cooking, playing music, playing with pets, playing chess and cards, etc.[16]. Watching television, videos or digital video discs (DVD) was classified as midrange activity and was not included as near work. Schoolwork during the class was not included in our questionannaire [16]. Outdoor activities included running, swimming, dancing, bicycle riding, playing football, table tennis, badminton and basketball, exercise between classes, throwing sandbags, skipping rope, rubber band skipping and kicking a shuttle cock, etc. The parents were asked specifically to differentiate the activities as indoors or outdoors.

To control interview bias from questionnaire survey, the staff were first trained for many times until that every question could be understood clearly and consistently by our staff. Secondly, the questionnaire was interviewer-administered which meant that any confusion from the parents about the questionnaire could be explained in time. Thirdly, we performed a pilot study on the validity and reliability of the questionnaire [19], and found that the questionnaire had an overall intraclass correlation coefficient of 0.63 between two repeated surveys (with an interval of 3 weeks), a Cronbach’s α coefficient for each item of 0.61, and load values of 12 items on common factors that were all greater than 0.4 [19]. Fourthly, the questionnaires were rechecked after return from the parents. The confusing answers would be identified by contacting the parents. Near work activity levels were divided into low, moderate and high using population tertiles of the average daily hours spent in near work (0.29~2.75 hours/day; 2.76~4.04 hours/day; 4.05+ hours/day).

### Data Analysis

Refraction was calculated as spherical equivalent (SE, sphere power+cylinder power/2). The mean right eye and left eye SE values were highly correlated (Pearson correlation coefficient = 0.92, P<0.0001). Thus, only right eyes were included in the analysis. We defined myopia as SE ≤ -0.50 D. Statistical analyses were performed using SAS (v9.3, SAS Institute Inc., Cary, NC, USA). First, we performed univariate analysis including general linear models between SE and one near work related parameter only, and logistic regression between the presence of myopia and one near work related parameter only. Second, we performed multivariate analysis with the SE and the presence of myopia as the dependent variables, with near work related parameters as independent variables that demonstrated significance in the univariate analysis, and with adjustment of age, gender, height and number of myopic parents. Interactions between near work related parameters and other risk factors for myopia were assessed in general linear models. Chi-square tests were used in categorical variables or in continuous variables by dividing them into categories, which has been used in previous study [16]. P≤0.05 was considered to be statistically significant. All P values were 2-sided. Odds ratios (OR’s) and 95% confidence intervals (95% CI) are presented.

## Results

Of the 2267 grade 7 students, 2101 were included after excluding those with ocular pathology such as amblyopia, strabismus or any other conditions that might affect the analysis. Of them, 1770 (78.1%) had available data for this study. Their mean age was 12.7 (range, 10~15) years and boys accounted for 47.9%. Mean SE was -1.6±2.1 D and mean axial length was 24.1±1.1 mm. There were no significant differences in age, number of children at each age, spherical equivalent, axial length, height, weight and number of myopic parents between children included and excluded from this study except that more girls were included (Table 1). Please not that some children might miss few variables and the numbers of children were slightly smaller than 1770 for some variables.

**Table 1:** Comparison of the participants that were included vs. excluded from the study.

Characteristics	Included (n = 1770)	Excluded (n = 331)	P
Age (mean±SD)	12.7±0.5	12.7±0.5	0.14
No. of each age (n, %)			0.30
10- years	1 (0.1%)	0 (0%)	
11- years	75 (4.2%)	15 (4.6%)	
12- years	1305 (73.8%)	232 (70.3%)	
13- years	361 (20.4%)	73 (22.1%)	
14- years	23 (1.3%)	9 (2.7%)	
15- years	3 (0.2%)	1 (0.3%)	
Gender (n, %)			<0.01
Girl	921 (52.1%)	141 (42.6%)	
Boy	848 (47.9%)	190 (57.4%)	
Spherical equivalent	-1.6±2.1	-1.5±2.0	0.41
Axial length	24.1±1.1	24.2±1.1	0.76
Visual acuity (LogMAR)	0.0±0.1	0.0±0.2	0.40
Height	154.9±7.0	155.2±7.7	0.56
Weight	47.5±10.0	48.4±10.7	0.16
Number of myopic parents (n, %)			0.79
None	1062 (69.9%)	178 (67.7%)	
One	365 (23.9%)	66 (25.1%)	
Two	98 (6.4%)	19 (7.2%)	

### Near work and Outdoor Activities by Gender and Age

The time spent in near work and outdoor activities had a weak positive correlation (r = 0.39). Completing homework, reading books for pleasure, using a computer and playing console games were major components of near work activities (Table 2). Boys spent more time in using a computer and in outdoor activities than girls. The older children spent more time in playing console games and less time in homework than the younger children.

**Table 2:** Time spent in near work and outdoor activities (hours/day) by gender and age.

	n	Completing Homework	Reading Books for Pleasure	Using a Computer	Playing Console Games	Combined Near work	Combined Outdoor Activities
All children	1547	1.72 (1.69–1.76)	0.77 (0.73–0.80)	0.64 (0.60–0.67)	0.41 (0.36–0.47)	3.70 (3.61–3.79)	2.08 (3.61–3.79)
Sex							
Girls	800	1.76 (1.71–1.81)	0.74 (0.70–0.79)	0.51 (0.47–0.55)	0.38 (0.30–0.46)	3.66 (3.54–3.79)	1.80 (1.72–1.88)
Boys	747	1.69 (1.63–1.74)	0.79 (0.74–0.85)	0.77 (0.71–0.83)	0.44 (0.37–0.51)	3.75 (3.61–3.88)	2.38 (2.27–2.50)
*P*		0.06	0.14	**<0.0001**	0.27	0.36	**<0.0001**
Age (years)							
12	1214	1.75 (1.70–1.79)	0.76 (0.73–0.80)	0.63 (0.59–0.67)	0.37 (0.31–0.43)	3.72 (3.62–3.82)	2.08 (2.00–2.16)
13	333	1.64 (1.56–1.72)	0.77 (0.69–0.85)	0.67 (0.59–0.74)	0.55 (0.42–0.68)	3.66 (3.45–3.87)	2.08 (1.92–2.24)
*P*		**0.03**	0.88	0.38	**0.01**	0.61	0.97

Bold values indicate statistical significance (P < 0.05).

### Near work Activity and Refraction

No associations were found between time spent in near work and refraction (P = 0.83) in this sample of Chinese children. Among different near work activities, only reading more books for pleasure was significantly associated with greater myopic refraction (P = 0.03, S1 Table).

### Near work Related Parameters, Refraction and Myopia

Continuous reading, television viewing distance, head tilt when writing, desk light, reading distance and nib-to-fingertip distance were significantly associated with mean SE in univariate analysis (Table 3). After adjusting for age, sex, height and number of myopic parents, these factors remained significant associations. The mean SE was more myopic with longer time spent in continuous reading (P = 0.009), closer television viewing distance (P<0.0001), head tilt when writing (P = 0.004), use of fluorescent desk light (P<0.001), closer reading distance (P = 0.0002) and closer nib-to-fingertip distance (P = 0.02).

**Table 3:** Associations of Near work Related Parameters with SE.

Near work Related Parameters(unit, number of subjects)	Mean SE (diopters, 95% CI)	
	Univariate Analysis	Multivariate Analysis Adjusted for Age, Sex, Number of Myopic Parents and Height
Continuous reading* (minutes, 1595)		
≤ 15	-1.27 (-1.68, -0.87)	-1.28 (-1.76, -0.80)
16–30	-1.28 (-1.50, -1.05)	-1.38 (-1.65, -1.11)
31–45	-1.54 (-1.72, -1.36)	-1.66 (-1.88, -1.44)
46–60	-1.71 (-1.93, -1.50)	-1.73 (-1.97, -1.48)
> 60	-1.86 (-2.08, -1.63)	-1.71 (-1.99, -1.44)
*P*	**0.002**	**0.009**
Television viewing distance (m, 1533)		
≤ 1	-2.44 (-2.99, -1.89)	-2.49 (-3.20, -1.79)
1–2	-1.84 (-2.01, -1.66)	-1.83 (-2.05, -1.62)
2–3	-1.46 (-1.61, -1.31)	-1.52 (-1.69, -1.34)
> 3	-1.14 (-1.39, -0.89)	-1.26 (-1.57, -0.95)
*P*	**<0.0001**	**<0.0001**
Head tilt when writing (1595)		
Yes	-1.78 (-1.96, -1.61)	-1.68 (-1.87, -1.49)
NO	-1.45 (-1.59, -1.31)	-1.55 (-1.71, -1.40)
*P*	**0.003**	**0.004**
Desk light (1749)		
Fluorescent lamp	-1.67 (-1.78, -1.56)	-1.68 (-1.81, -1.54)
Incandescent lamp	-1.27 (-1.49, -1.05)	-1.37 (-1.62, -1.12)
*P*	**<0.001**	**<0.001**
Reading distance (cm,1452)		
≤ 10	-2.12 (-2.53, -1.71)	-1.93 (-2.53, -1.71)
10–20	-1.78 (-1.94, -1.61)	-1.78 (-1.94, -1.61)
20–30	-1.45 (-1.62, -1.29)	-1.45 (-1.62, -1.29)
> 30	-1.09 (-1.42, -0.76)	-1.09 (-1.42, -0.76)
*P*	**<0.0001**	**0.0002**
Nib to finger distance (cm, 1595)		
≤ 2	-1.70 (-1.83, -1.57)	-1.69 (-1.83, -1.56)
> 2	-1.41 (-1.59, -1.24)	-1.42 (-1.60, -1.25)
*P*	**0.01**	**0.02**
Night-lights (1627)		
Yes	-1.63 (-1.94, -1.33)	-1.58 (-1.87, -1.29)
No	-1.58 (-1.69, -1.47)	-1.58 (-1.69, -1.48)
*P*	0.73	0.98

*Defined as time spent in continuous reading before taking a break of 5 minutes or longer;

Bold values indicate statistical significance (P < 0.05).

Children were significantly more likely to be myopic when they were reported to have continuous reading time >45 min (OR,1.4; 95% CI, 1.1~1.8), television viewing distance ≤3 m (OR, 1.7; 95% CI, 1.2~2.3), head tilt when writing (OR, 1.3; 95% CI, 1.1~1.7), and use of fluorescent desk lamp (OR, 1.5; 95% CI, 1.2~2.0) (Table 4). There was borderline significance between nib-to-fingertip distance and the presence of myopia (P = 0.07).

**Table 4:** Associations of Near work Related Parameters with the Presence of Myopia and Axial Length.

Near work Related Parameters (unit, number of subjects)	The Presence of Myopia, OR (95% CI)		Axial Length, (mm, 95% CI)	
	Univariate Analysis	Multivariate Analysis Adjusted for Age, Sex, Number of Myopic Parents and Height	Univariate Analysis	Multivariate AnalysisAdjusted for Age, Sex, Number of Myopic Parents and Height
Continuous reading* (minutes, 1595)				
≤ 45	1 (Reference)	1 (Reference)	24.08 (24.01–24.15)	24.10 (24.04–24.17)
> 45	1.49 (1.20, 1.85)	1.40 (1.09, 1.80)	24.21 (24.13–24.30)	24.18 (24.10–24.25)
*P*	**0.0003**	**0.008**	**0.02**	0.18
Television viewing distance (m, 1533)				
> 3	1 (Reference)	1 (Reference)	23.93 (23.79–24.06)	23.92 (23.79–24.05)
≤ 3	1.74 (1.32, 2.30)	1.67 (1.23, 2.28)	24.18 (24.12–24.24)	24.18 (24.13–24.24)
*P*	**<0.0001**	**0.001**	**0.0007**	**0.0002**
Head tilt when writing (1595)				
Yes	1.49 (1.19, 1.88)	1.32 (1.03, 1.70)	24.17 (24.08–24.25)	24.20 (24.11–24.28)
No	1 (Reference)	1 (Reference)	24.12 (24.04–24.19)	24.10 (24.03–24.17)
*P*	**0.0007**	**0.03**	0.41	0.08
Desk light (1749)				
Fluorescent lamp	1.53 (1.21, 1.93)	1.53 (1.17, 2.00)	24.20 (24.14–24.26)	24.19 (24.13–24.09)
Incandescent lamp	1 (Reference)	1 (Reference)	23.96 (23.86–24.07)	23.99 (23.90–24.09)
*P*	**0.0003**	**0.002**	**0.0001**	**0.0006**
Reading distance (cm,1452)				
≤ 20	1.23 (0.99, 1.54)	0.96 (0.75, 1.24)	24.23 (24.15–24.30)	24.23 (24.15–24.30)
> 20	1 (Reference)	1 (Reference)	24.10 (24.02–24.18)	24.10 (24.02–24.17)
*P*	0.06	0.77	**0.02**	**0.01**
Nib to finger distance (cm, 1595)				
≤ 2	1.26 (1.01–1.58)	1.25 (0.99, 1.59)	24.23 (24.16–24.29)	24.22 (24.15–24.29)
> 2	1 (Reference)	1 (Reference)	24.01 (23.92–24.10)	24.01 (23.92–24.10)
*P*	**0.04**	0.07	**0.0002**	**0.002**
Night-lights (1627)				
Yes	1 (Reference)	1 (Reference)	24.11 (23.96–24.25)	24.15 (24.00–24.29)
No	0.88 (0.63, 1.23)	0.88 (0.57, 1.35)	24.15 (24.09–24.21)	24.15 (24.09–24.20)
*P*	0.46	0.56	0.64	0.99
Combined Near work (hours/day, 1770)				
< 2.75	1 (Reference)	1 (Reference)	24.14 (24.06–24.22)	24.16 (24.08–24.23)
2.75–4.04	0.99 (0.78, 1.26)	1.10 (0.81, 1.49)	24.13 (24.04–24.22)	24.14 (24.05–24.23)
> 4.04	1.02 (0.80, 1.30)	0.89 (0.65, 1.20)	24.16 (24.07–24.25)	24.12 (24.04–24.21)
*P*	0.94, 0.87	0.55, 0.43	0.92	0.85

*Defined as time spent in continuous reading before taking a break of 5 minutes or longer;

Bold values indicate statistical significance (P < 0.05).

Children who spent greater time in reading books reported longer periods of continuous reading (39.7%, 40.6% and 47.1%, χ^2^ = 0.04). Children who reported closer nib-to-fingertip distance had significantly greater chance of head tilt when writing (43.5% vs. 36.7%, χ^2^ = 0.02). Children using a fluorescent desk light had more myopic parents (χ^2^ = 0.001). There was no association, however, between time in reading books and reading distance (χ^2^ = 0.98), between reading books and nib-to-fingertip distance (χ^2^ = 0.28), or between continuous reading and reading distance (χ^2^ = 0.66).

### Near work Related Parameters and Axial Length

In multivariate models, children were significantly more likely to have longer axial length of 0.26 mm, 0.20 mm, 0.13 mm and 0.21 mm, respectively, with television viewing distances ≤3 m, fluorescent desk lamp use, reading distances <20 cm and nib-to-fingertip distance <2 cm. There was a borderline significant association for head tilt when writing (P = 0.08) (Table 4).

### Interaction of Near work Related Parameters and Parental Myopia

**Fig 1:**
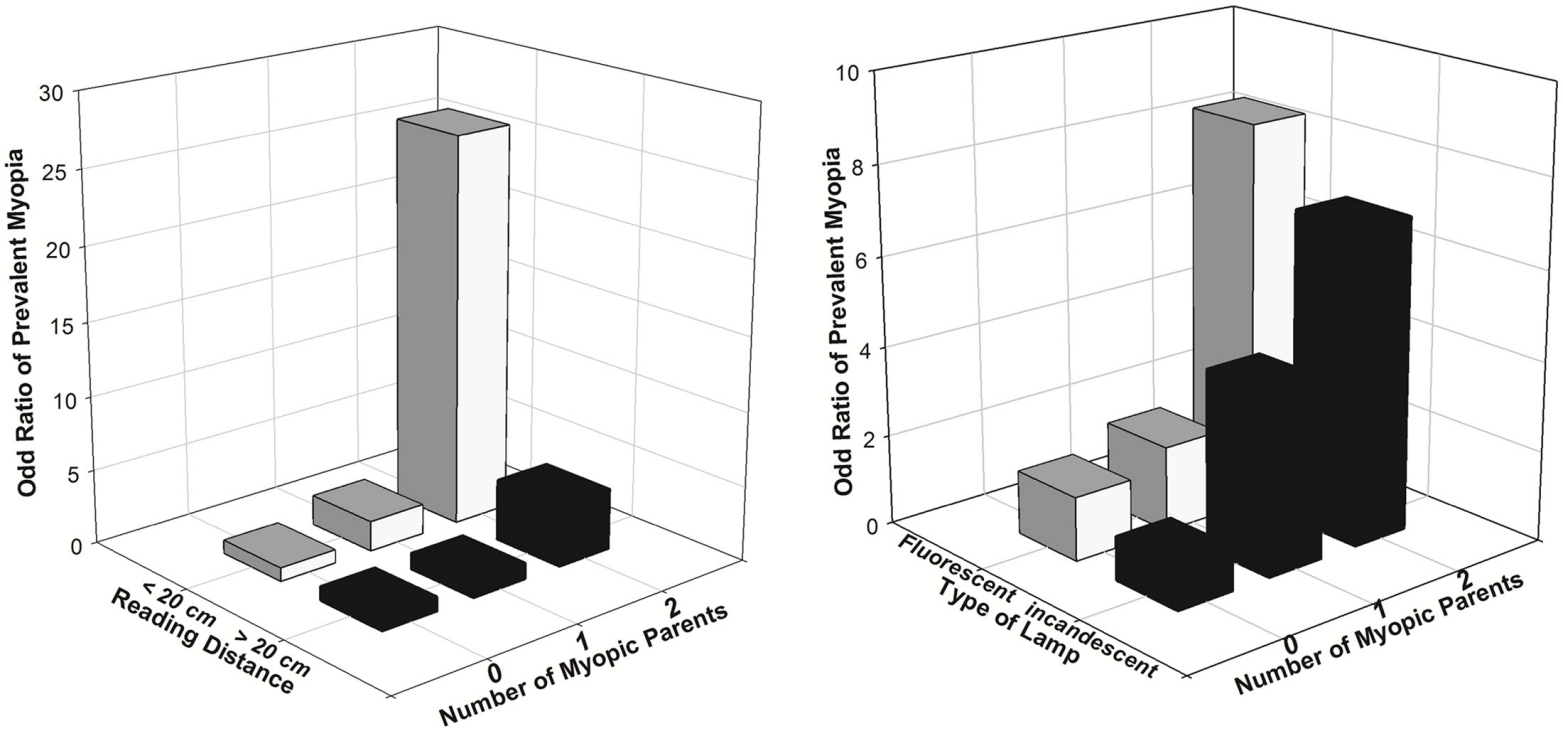
Multivariable-adjusted odds ratios (OR, adjusted for age, gender and height) for prevalent myopia by reading distance versus number of myopic parents (1A) and by type of desk light versus number of myopic parents (1B) in children. Reading distance was divided into ≤ 20 cm and > 20 cm. The children with reading distance > 20 cm and no myopic parents is the reference group (OR, 1). Type of desk light was divided into fluorescent and incandescent lamp. The children with desk light of incandescent lamp and no myopic parents is the reference group (OR, 1).

In multivariate models, there was a significant interaction of parental myopia and close reading distance for a greater likelihood of myopia (P = 0.02). Children with close reading distances and two myopic parents had 26-fold higher odds for prevalent myopia (OR, 26.3; 95% CI, 3.6–191.1, P = 0.001) than children with reading distances > 20 cm and no myopic parent (Fig 1A). The number of children with 0, 1 and 2 myopic parents was 438, 184 and 56 for those with close reading distance, and 516, 149 and 36 for those with reading distances > 20 cm.

The interaction effect from parental myopia and fluorescent desk light use was significant for increasing myopic refraction (P = 0.02) and greater likelihood of myopia (P = 0.0008). Children using fluorescent desk lamps and with two myopic parents had 8-fold higher odds for prevalent myopia (OR, 8.6; 95% CI, 3.7–20.1, P<0.0001) than children who used incandescent desk lights and did not have a myopic parent (Fig 1B). The number of children with 0, 1 and 2 myopic parents was 276, 69 and 13 for those with incandescent lamp, and 776, 289 and 85 for those with fluorescent lamp.

**Fig 2:**
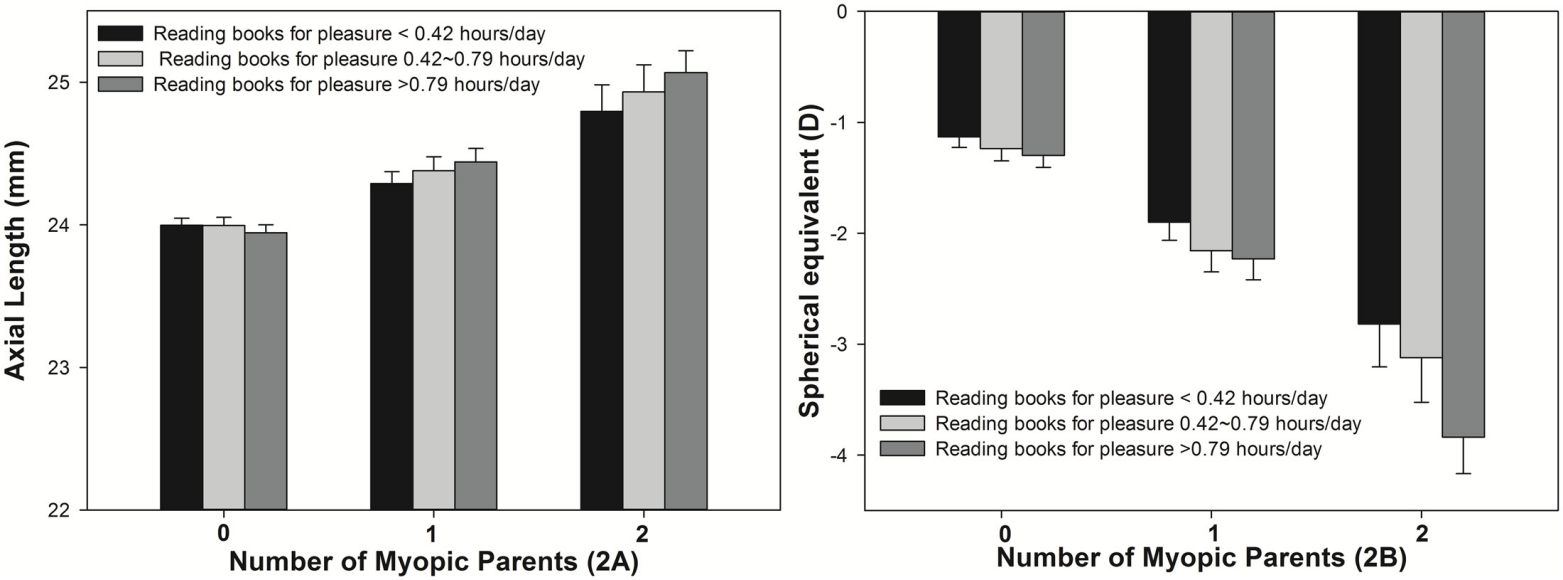
Age-gender-height adjusted axial lengths (2A, mm, mean±SEM, P = 0.04) and spherical equivalent (2B, D, mean±SEM, P = 0.05) by reading books for pleasure per week and parental myopia using general linear model.

The interaction effect from parental myopia and a history of reading more books for pleasure was statistically significant for longer axial length values (P = 0.04) and of borderline significance for SE (P = 0.05) (Fig 2A and 2B). Children with two myopic parents and who read books for more than 0.79 hours/day (highest category) had 1.1 mm longer axial length and 2.7D greater myopia than children with no myopic parent and who read books less than 0.42 hours/day (lowest category).

## Discussion

In this study with a large sample size of 12-year-old Chinese children, we confirmed that continuous reading and close reading distance were significantly associated with myopia, a finding previously reported in 12-year-old Caucasian children but not in 12-year-old East Asian children in Australia [16]. More importantly, we found that closer television viewing distance, head tilt when writing, use of fluorescent desk lighting and closer nib-to-fingertip distance were also significantly associated with greater myopia and longer axial length.

Consistent with previous study [16], time spent in near work was not associated with myopia in this study. By comparison, our children had similar level of near work (25.9 vs. 27.4 hours/week) and outdoor activities (14.6 vs. 12.3 hours/week), but significantly higher myopia prevalence (67.3% vs. 12.8%) and more myopic refraction (-1.57D vs. +0.49D) than theirs [16]. These differences could not be explained by the time in near work and outdoor activities. Note that our children had spent almost twice the time in homework as theirs (12.1 vs. 7.6 hours/week) (Table 2) [16]. This significant difference, combined with the evidence that emmetropic schoolchildren in China usually have reading behaviors such as close reading distance and head tilt [17], led to the idea that some reading and writing behaviors during near work, which were evaluated in this study, could have contributed to the development of myopia.

If East Asian children at both sites were selected controlling for the influence of ethnic difference, the Chinese children in this study still had considerably higher myopia prevalence (67.3% vs.41.6%) and greater myopic levels (mean -1.57D vs. -0.5D) than in the Sydney cohort [20]. Comparing environmental factors, our children had more time in outdoors (14.6 vs. 8.5 hours/week), less time in near work (25.9 vs. 32.5 hours/week), but more time in homework (12.1 vs. 9.1 hours/week) than the East Asian children in the Sydney cohort [16]. Therefore, near work related parameters were more likely to play an important role in myopia.

In our study, only reading more books for pleasure was associated with more myopia. Further, interaction between parental myopia and reading books for pleasure was also found. These findings were consistent with those of Singapore Chinese children [6]. Moreover, we found an interaction between parental myopia and close reading distance. It is likely that behaviors during reading contributed to this association. However, it remains unclear whether children with myopic parents may inherit a susceptibility to certain reading and writing behaviors, or whether these are simply a consequence of following parental reading behaviors, close reading distance, continuous reading and others.

Consistent with previous report [16], our children with more time in reading had longer period of continuous reading. Further, during longer periods of continuous reading, children may be likely to take up their preferred relaxed postures [21], such as a head tilt or reading in bed with a close reading distance. In addition, we found that children with closer nib-to-fingertip distance had greater head tilt when writing. Head tilt, however, may cause higher dioptric stimuli, abnormal contractions of extraocular muscles and markedly out-of focus in the peripheral retina [21], which could be associated with myopia.

Watching television is usually considered as a mid-distance visual activity. Previous studies on the association between watching television and myopia have been controversial [2,8,22]. You et al. reported an association of myopic refraction with longer duration of watching television in Greater Beijing school children [23], whereas Parssinen et al. reported that watching television > 3 hours per day was a protective factor for greater myopia [22]. You et al. also found an association of television viewing distance and myopia in univariate analysis, but this was not significant in multivariate analysis [23]. In our study, children with closer television viewing distance (≤3 m) had greater myopia in both the univariate and multivariate analysis. These findings indicate that the association between watching television and myopia may be distance-dependent, and in general, the impact of television on myopia is lower [8].

Interestingly, we found that using a fluorescent desk lamp was associated with myopic refraction and axial length. In out study, most families were still using a traditional fluorescent lamp with low frequency of flicker, which has been reported to be capable of inducing myopia in mice [24]. In addition, low light levels[25] and the narrow light spectrum of fluorescent lamps could also contribute. To the contrary, incandescent lamps provide relatively higher light levels and a wider light spectrum which is relatively close to that of outdoors [26]. Czepita et al. reported that the use of fluorescent lamps before age two was associated with an increase in the occurrence of astigmatism [27]. We only asked about use of fluorescent lamps around age 12, not at such an early age. We also found that children who used fluorescent lamps had significantly more myopic parents with an interaction also found between fluorescent desk lamp use and parental myopia. Whether this association attributes to the lamp characteristic, parental myopia, or both, remains unclear. In our study, there was no association between use of night-lights and myopia in accordance with previous studies [28].

Walline et al.[29] have summarized that the most effective treatment to slow myopia progression thus far is atropine eyedrops. Combining the findings in the present study, the mechanisms of atropine eyedrops on slowing myopia progression may be related to its cycloplegic effect which could lead to farther distance on reading, writing and watching TV, less near work such as reading, and be related to its dilated pupil size which could lead to more entrance of sunlight. These hypotheses need further evaluation in further study.

Some limitations of this study should be mentioned. First, data on near work and its related parameters were obtained from questionnaires rather than direct measurements, which could have led to recall bias. Although we have used interviewer-administered survey to clarify the confusion of parents in time, and have performed a pilot study with a better repeatability, recall bias might still cause misunderstanding of these associations. The novel instrument for logging near work distance [30] or other factors is more accurate and would be expected to be used in future study. Second, all near work related parameters and myopia were measured at only one time point in this study, so we cannot conclude that there are any cause–effect relationships. These various reading and writing habits might be simply the results of myopia. Third, the validity of some scales in this study has not been reported previously. The use of a variable number of bins may affect the sensitivity of different measures to a different extent. These need caution when interpreting these results in other conditions.

In conclusion, our study showed that near work related modifiable factors such as continuous reading, close reading distance, close television viewing distance, head tilt when writing, use of fluorescent desk lamps and close nib-to-fingertip distance were associated with myopia in Chinese children. Future trials of intervention on these factors, for example, timely break of near work, reading and watching at a longer distance and more healthy writing habits may be important for controlling myopia.

## Supporting Information

S1 File Questionnaire used in this study. (DOC)

S1 Table Associations of Nearwork Activities with SER in Children. (DOC)
