# Supplementary material for: Near Work Related Parameters and Myopia in Chinese Children: the Anyang Childhood Eye Study
Source: PLoS One. 2015 Aug 5;10(8):e0134514. doi: 10.1371/journal.pone.0134514 (PMC4526691; doi:10.1371/journal.pone.0134514)
Supplement: S1 File — (DOC) [file pone.0134514.s001.doc]

Supplementary file

1. Questions on nearwork

| **Items** |  | |
| --- | --- | --- |
| 1. Homework | _______time/week | ______hours _____minutes for each time |
| 1. Reading books for pleasure | _______time/week | ______hours _____minutes for each time |
| 1. Video/ DVD | _______time/week | ______hours _____minutes for each time |
| 1. Playing Console Games | _______time/week | ______hours _____minutes for each time |
| 1. drawing, painting, writing | _______time/week | ______hours _____minutes for each time |
| 1. cooking | _______time/week | ______hours _____minutes for each time |
| 1. playing music | _______time/week | ______hours _____minutes for each time |
| 1. playing with pets | _______time/week | ______hours _____minutes for each time |
| 1. playing chess and cards | _______time/week | ______hours _____minutes for each time |
| 1. Others, please describe____ | _______time/week | ______hours _____minutes for each time |
| 1. Others, please describe____ | _______time/week | ______hours _____minutes for each time |
| 1. Others, please describe____ | _______time/week | ______hours _____minutes for each time |

1. Questions on outdoor activity

| **Items** |  | | **OUTDOOR** | **INDOOR** |
| --- | --- | --- | --- | --- |
| 1. Running | _______time/week | ______hours _____minutes for each time |  |  |
| 1. Swimming | _______time/week | ______hours _____minutes for each time |  |  |
| 1. Playing football | _______time/week | ______hours _____minutes for each time |  |  |
| 1. Playing badminton | _______time/week | ______hours _____minutes for each time |  |  |
| 1. Playing table tennis | _______time/week | ______hours _____minutes for each time |  |  |
| 1. Playing basketball | _______time/week | ______hours _____minutes for each time |  |  |
| 1. Exercise between classes | _______time/week | ______hours _____minutes for each time |  |  |
| 1. Playing games (throwing sandbags, skipping rope, rubber band skipping and kicking a shuttle cock) | _______time/week | ______hours _____minutes for each time |  |  |
| 1. Dancing | _______time/week | ______hours _____minutes for each time |  |  |
| 1. Bicycle riding | _______time/week | ______hours _____minutes for each time |  |  |
| 1. Others, please describe____ | _______time/week | ______hours _____minutes for each time |  |  |
| 1. Others, please describe____ | _______time/week | ______hours _____minutes for each time |  |  |

1. Please evaluate the distance between book and child’s face when your child read or write

□ 0-10 cm □ 10-20 cm □ 20-30 cm □ >30 cm □ Not sure

1. Does your child has the habit of head tilt when writing?

□ No □ Yes □ Not sure

1. Please evaluate the distance between nib to finger when your child write.

□ <2cm □ >2 cm □ Not sure

1. Please evaluate the distance between TV and child when he/she watch TV

□ <1 m □ 1-2 m □ 2-3 m □ > 3m □ Not sure

1. Does your child use desk light when he/she read or doing nearwork

□ No □ Yes □ Not sure

If the desk light is used, which type is the bulb?

□ Fluorescent lamp □ Incandescent lamp □ Not sure

1. Does your child has the haibt of turn on night lights when sleep?

□ No □ Yes □ Not sure

1. Usually, how long will your child continuously read or doing nearwork before he/she has a rest ?

□ 0-15 minutes

□ 16-30 minutes

□ 31-45 minutes

□ 46-60 minutes

□ 61-90 minutes

□ 91-120 minutes

□ >120 minutes
