# Supplementary material for: Near Work Related Parameters and Myopia in Chinese Children: the Anyang Childhood Eye Study
Source: PLoS One. 2015 Aug 5;10(8):e0134514. doi: 10.1371/journal.pone.0134514 (PMC4526691; doi:10.1371/journal.pone.0134514)
Supplement: S1 Table — (DOC) [file pone.0134514.s002.doc]

Supplementary Table . Associations of Nearwork Activities with SER in 12-year-old Children

| **Nearwork Activities (hours/day)** | **Mean SER (95% CI)** | |
| --- | --- | --- |
|  | **Univariate Analysis** | **Multivariate Analysis**  **Adjusted for Age, Sex, Number of Myopic Parents and Height** |
| School homework* |  |  |
| < 1.29 | -1.49 (-1.65, -1.32) | -1.50 (-1.67, -1.33) |
| 1.29-1.96 | -1.67 (-1.83, -1.51) | -1.68 (-1.84, -1.52) |
| > 1.96 | -1.55 (-1.72, -1.39) | -1.52 (-1.70, -1.35) |
| *P* | 0.28 | 0.21 |
| Reading books for pleasure |  |  |
| < 0.42 | -1.44 (-1.59, -1.29) | -1.47 (-1.62, -1.32) |
| 0.42-0.79 | -1.55 (-1.73, -1.38) | -1.56 (-1.73, -1.39) |
| > 0.79 | -1.74 (-1.92, -1.57) | -1.69 (-1.86, -1.53) |
| *P* | **0.03** | **0.03** |
| Using a computer |  |  |
| < 0.29 | -1.67 (-1.82, -1.53) | -1.64 (-1.78, -1.49) |
| 0.29-0.64 | -1.50 (-1.68, -1.32) | -1.50 (-1.66, -1.33) |
| > 0.64 | -1.47 (-1.65, -1.28) | -1.54 (-1.72, -1.35) |
| *P* | 0.16 | 0.18 |
| Playing console games |  |  |
| < 0.15 | -1.59 (-1.70, -1.48) | -1.59 (-1.69, -1.48) |
| 0.15-0.36 | -1.45 (-1.75, -1.16) | -1.45 (-1.76, -1.14) |
| > 0.36 | -1.52 (-1.82, -1.21) | -1.52 (-1.84, -1.21) |
| *P* | 0.68 | 0.69 |
| Combined Nearwork |  |  |
| < 2.75 | -1.55 (-1.70, -1.41) | -1.56 (-1.70, -1.42) |
| 2.75-4.04 | -1.61 (-1.79, -1.43) | -1.62 (-1.79, -1.45) |
| > 4.04 | -1.56 (-1.73, -1.38) | -1.53 (-1.70, -1.36) |
| *P* | 0.88 | 0.83 |

Bold values indicate statistical signiﬁcance (P < 0.05)
